# Uncinate Fasciculus Lesion Burden and Anxiety in Multiple Sclerosis

**DOI:** 10.1001/jamanetworkopen.2025.4751

**Published:** 2025-04-14

**Authors:** Erica B. Baller, Audrey C. Luo, Matthew K. Schindler, Elena C. Cooper, Margaret K. Pecsok, Matthew C. Cieslak, Melissa Lynne Martin, Amit Bar-Or, Ameena Elahi, Christopher M. Perrone, Bailey C. Spangler, Theodore D. Satterthwaite, Russell T. Shinohara

**Affiliations:** 1 Department of Psychiatry, University of Pennsylvania, Philadelphia; 2 Penn Lifespan Informatics and Neuroimaging Center, Philadelphia, Pennsylvania; 3 Department of Neurology, University of Pennsylvania, Philadelphia; 4 Center for Neuroinflammation and Neurotherapeutics, University of Pennsylvania, Philadelphia; 5 Penn Statistics in Imaging and Visualization Center, Department of Biostatistics, Epidemiology, and Informatics, University of Pennsylvania, Philadelphia; 6 Department of Information Services, University of Pennsylvania, Philadelphia; 7 Center for Biomedical Image Computing and Analytics, University of Pennsylvania, Philadelphia

## Abstract

**Importance:**

Multiple sclerosis (MS) is an immune-mediated neurological disorder that affects 2.4 million people worldwide, and up to 60% experience anxiety.

**Objective:**

To investigate whether anxiety in MS is associated with white matter lesion burden in the uncinate fasciculus (UF).

**Design, Setting, and Participants:**

This was a retrospective case-control study of participants aged 18 years or older diagnosed with MS by an MS specialist and identified from the electronic medical record at a single-center academic medical specialty MS clinic in Pennsylvania. Participants received research-quality 3-Tesla magnetic resonance neuroimaging as part of MS clinical care from January 6, 2010, to February 14, 2018. After excluding participants with poor image quality, participants were stratified into 3 groups naturally balanced in age and sex: (1) MS without anxiety, (2) MS with mild anxiety, and (3) MS with severe anxiety. Analyses were performed from June 1 to September 30, 2024.

**Exposure:**

Anxiety diagnosis and anxiolytic medication.

**Main Outcomes and Measures:**

Main outcomes were whether patients with severe anxiety had greater lesion burden in the UF than those without anxiety and whether higher anxiety severity was associated with greater UF lesion burden. Generalized additive models were used, with the burden of lesions (eg, proportion of fascicle impacted) within the UF as the outcome measure and sex, spline of age, and total brain volume as covariates.

**Results:**

Among 372 patients with MS (mean [SD] age, 47.7 [11.4] years; 296 [80%] female), after anxiety phenotype stratification, 99 (27%) had no anxiety (mean [SD] age, 49.4 [11.7] years; 74 [75%] female), 249 (67%) had mild anxiety (mean [SD] age, 47.1 [11.1] years; 203 [82%] female), and 24 (6%) had severe anxiety (mean [SD] age, 47.0 [12.2] years; 19 [79%] female). UF burden was higher in patients with severe anxiety compared with no anxiety (*T* = 2.01 [*P* = .047]; Cohen *f*^2^, 0.19 [95% CI, 0.08-0.52]). Additionally, higher mean UF burden was associated with higher severity of anxiety (*T* = 2.09 [*P* = .04]; Cohen *f*^2^, 0.10 [95% CI, 0.05-0.21]).

**Conclusions and Relevance:**

In this case-control study of UF lesion burden and anxiety in MS, overall lesion burden in the UF was associated with the presence and severity of anxiety. Future studies linking white matter lesion burden in the UF with treatment prognosis are warranted.

## Key Points

**Question:**

Are white matter lesions that impact the uncinate fasciculus (UF) associated with anxiety in patients with multiple sclerosis (MS)?

**Findings:**

In this case-control study of 372 patients with MS stratified as without anxiety (n = 99), with mild anxiety (n = 249), or with severe anxiety (n = 24), patients with severe anxiety had higher UF lesion burden than those without anxiety, and worsening anxiety severity was associated with greater UF burden.

**Meaning:**

The findings suggest that lesion burden in the UF may be associated with anxiety comorbidity in MS.

## Teaser

This case-control study assesses the association of white matter lesion burden in the uncinate fasciculus with anxiety in patients with MS.

## Introduction

Multiple sclerosis (MS) is an immune-mediated neurological disorder that affects 2.4 million people worldwide.^1,2,3^ The rates of anxiety in MS are 3 times as high as in the general population, with up to 60% of persons with MS experiencing anxiety symptoms over their lifetime.^4^ Though anxiety in MS is associated with substantial disability, including worse physical functioning and quality of life,^4,5,6^ the mechanisms of anxiety in MS are not well understood.^5^ The few studies that have attempted to relate neuroinflammation and white matter pathology to anxiety in MS have not identified a reliable biomarker that directly links anxiety symptoms to brain pathology, leading some scientists to speculate that anxiety is a consequence of living with an unpredictable illness.^7,8,9,10^ However, 40% of those with MS do not have anxiety, suggesting that neural pathophysiology may contribute to anxiety risk. In this study, we evaluated whether lesions affecting white matter tracts that connect brain regions known to be associated with anxiety circuitry are associated with anxiety in MS.

Preclinical studies in rodents and primates have consistently demonstrated the importance of the orbitofrontal cortex (OFC) and the amygdala in anxiety and stress circuitry.^11,12,13,14^ Human neuroimaging studies have also shown convergent results.^15,16^ Across anxiety disorders, patients with anxiety show low OFC activation and high amygdala activation in functional magnetic resonance imaging (MRI).^17,18,19^ A study of anxiety in MS also identified abnormalities in resting state activity in the prefrontal cortex and amygdala.^20^ The prevailing hypothesis is that the OFC provides top-down control over the amygdala in healthy brain states and that disruption of that relationship leads to heightened amygdala activity, increased fear response, and subsequent activation of the sympathetic nervous system.^18,21^ The OFC and amygdala are structurally connected via the uncinate fasciculus (UF), and this structural relationship is conserved across species.^22,23^ Though lesions to the UF could theoretically lead to OFC and amygdala decoupling and cause anxiety, it is not possible to prospectively lesion the UF to test this hypothesis in humans.

Scientists have attempted to address this limitation by using diffusion imaging to study white matter variability between people with and without anxiety, but findings have been inconsistent. Though some studies have described associations between anxiety disorders and decreased fractional anisotropy in fascicles connecting the anterior and posterior cingulate, amygdala, and prefrontal cortex,^17,24,25,26^ others have not identified these associations.^27^ Additionally, nearly all studies have excluded participants with brain diseases and therefore cannot be extrapolated to MS. To advance our understanding of neuroanatomic vulnerability to anxiety in MS and to better characterize the pathophysiology of anxiety, studies with large sample sizes that examine the association of MS lesion location and burden with anxiety are necessary.

Herein, we assessed the association between anxiety and white matter lesion burden in the UF in a large sample of patients with MS who received research-quality structural imaging as part of routine care. We hypothesized that patients with MS who had a severe anxiety phenotype would have higher UF lesion burden than comparators who did not have anxiety and that anxiety severity would be associated with worse UF disease.

## Methods

### Participants

In this retrospective case-control study, participants with MS were identified from the electronic medical record (EMR) via the University of Pennsylvania Data and Analytics Center, including demographics; *International Statistical Classification of Diseases and Related Health Problems, 10th Revision (ICD-10)* diagnoses^28^; medications; and the Patient Health Questionnaire 2- and 9-question forms (PHQ-2 and PHQ-9).^29^ The University of Pennsylvania institutional review board approved this study and waived the requirement for informed consent due to the retrospective nature of the analysis. The study adhered to the Strengthening the Reporting of Observational Studies in Epidemiology (STROBE) reporting guideline.

Race was included because MS has historically been thought to primarily affect White individuals and may be underdiagnosed in individuals of other races. Patients self-reported their race; categories were American Indian or Alaskan Native, Asian, Black or African American, East Indian, Hispanic Latino Black, Hispanic Latino White, Native Hawaiian or Other Pacific Islander, White, and other. Unknown race or those who declined to answer were included as separate categories.

### MS Diagnosis

Participants aged 18 years or older were included if they received an *ICD-10* MS diagnosis (G35) from a specialist in the Penn MS and Related Disorders Center and received 3-Tesla (3T) MRI under the University of Pennsylvania MS protocol^30^ from January 6, 2010, to February 14, 2018. Because no current biomarkers reliably distinguish between MS subtypes, all MS subtypes were considered together.^31^

### Anxiety Diagnosis and Severity Stratification

**Figure 1.**
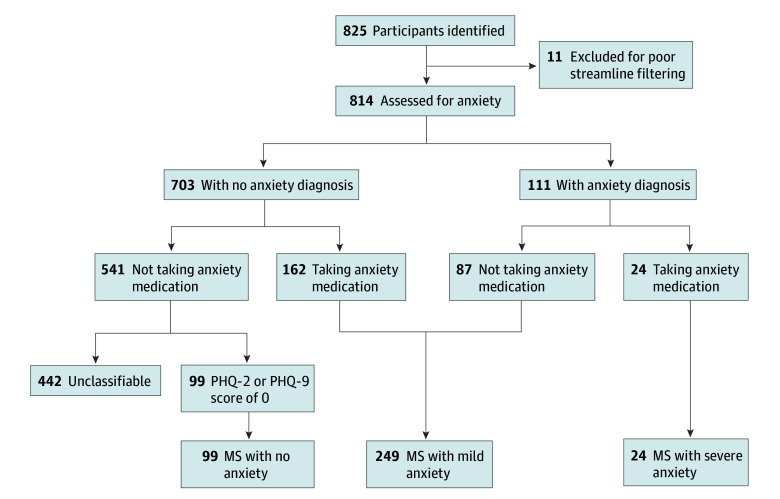
Flowchart of Study Population Phenotyping MS indicates multiple sclerosis; PHQ-2, 2-question Patient Health Questionnaire; PHQ-9, 9-question Patient Health Questionnaire.

It is well known that anxiety is underdiagnosed and undertreated in medical populations, which creates challenges for capturing anxiety phenotypes from the EMR.^32,33,34^ We developed an anxiety stratification method that integrated data from 3 fields: *ICD-10* diagnoses, medication prescriptions, and PHQ ratings (Figure 1).

Participants with MS were stratified into 3 groups: (1) MS without anxiety, (2) MS with mild anxiety, and (3) MS with severe anxiety. MS without anxiety included persons who had no psychiatric diagnoses, took no psychiatric medications, and were asymptomatic based on the PHQ-2 or PHQ-9 (score = 0). To classify MS with mild or severe anxiety, we made 2 assumptions based on known physician undercoding behavior.^35,36,37^ We assumed that the presence of either an anxiety diagnosis (*ICD-10* code F40*) or an anxiolytic medication (eTable 1 in Supplement 1) suggested some anxiety symptom burden (MS with mild anxiety), whereas the presence of both an anxiety diagnosis and an anxiety medication was assumed to indicate that psychopathology was severe enough to be diagnosed and treated pharmacologically (MS with severe anxiety).^38^ Participants without psychiatric diagnoses or medications were excluded from the group without anxiety if no PHQ-2 or PHQ-9 scores were available to confirm the absence of psychopathology. For all analyses, we defined *anxiety diagnosis* as a comparison between MS without anxiety and MS with severe anxiety and *anxiety severity* as a parametric comparison across all 3 groups (no anxiety, mild anxiety, and severe anxiety).

### PROMIS Validation

Given the assumptions required for group stratification, we next tested whether our anxiety diagnosis and severity stratification captured phenotypic variation in functioning. To do this, we evaluated group differences in Patient-Reported Outcomes Measurement Information System (PROMIS) scores in a subset of patients, which has been validated in MS.^39,40^ PROMIS assesses current symptom burden in 10 domains, including mental health and mood, emotional problems, quality of life, physical health, satisfaction with social activities, carrying out social activities, carrying out physical activities, fatigue, overall physical impairment, and overall mental impairment. Because PROMIS was not used for anxiety stratification, it served as an independent measure of our phenotyping algorithm. For patients with multiple PROMIS scores, the score most proximal to their MRI was used.

We next calculated 2 summary measures: a physical functioning score (mean of PROMIS scores for physical health, carrying out physical activities, fatigue, and overall physical impairment) and an emotional functioning score (mean of PROMIS scores for mental health and mood, emotional problems, quality of life, satisfaction with social activities, carrying out social activities, and overall mental impairment). Quality of life was included in emotional functioning given prior work suggesting that it better correlates with psychiatric vs physical impairment.^41,42,43,44^ Finally, we tested whether our EMR-derived anxiety phenotypes were differentially associated with physical and emotional functioning.

### Image Acquisition and Processing

Structural 3T MRI was obtained as part of routine care using a research-quality protocol, including 3-dimensional (3D) T1-weighted magnetization-prepared rapid gradient echo imaging (repetition time [TR] = 1.9 seconds, time to echo [TE] = 2.48 milliseconds, inversion time [TI] = 900 milliseconds, flip angle [FA] = 9°, acquisition time = 4:18, 176 sagittal slices, and resolution = 1 mm^3^) and 3D T2-weighted fluid-attenuated inversion recovery (FLAIR) (TR = 5 seconds, TE = 398 milliseconds, TI = 1.8 seconds, FA = 120°, acquisition time = 5:02, 160 sagittal slices, and resolution = 1 mm^3^). Images were processed using a top-performing pipeline as previously described.^30^ In brief, T1-weighted and FLAIR images were N4-bias corrected,^45^ extracerebral voxels were removed from the T1-weighted images using multi-atlas skull stripping,^46^ T1-weighted images and their corresponding brain masks were registered to the corresponding FLAIR image, and skull-stripped FLAIR and aligned T1-weighted images were intensity normalized using WhiteStripe.^47^ T1-weighted images were segmented with the Functional Magnetic Resonance Imaging of the Brain automated segmentation tool, and total brain volume was calculated by summing gray and white matter volumes.^48,49^ MRI was usually acquired within 6 months of presentation to the MS clinic. For participants with multiple scans, their first clinical MRI was used.

### Automated Lesion Segmentation and Streamline Filtering

We performed fully automated lesion segmentation with the Method for Intermodal Segmentation Analysis (MIMoSA) to obtain binary maps of white matter lesions in a participant’s native space.^50^ The quality of processed images and segmentations was assessed by a scientist (M.L.M.) with years of experience in MS imaging research.

**Figure 2.**
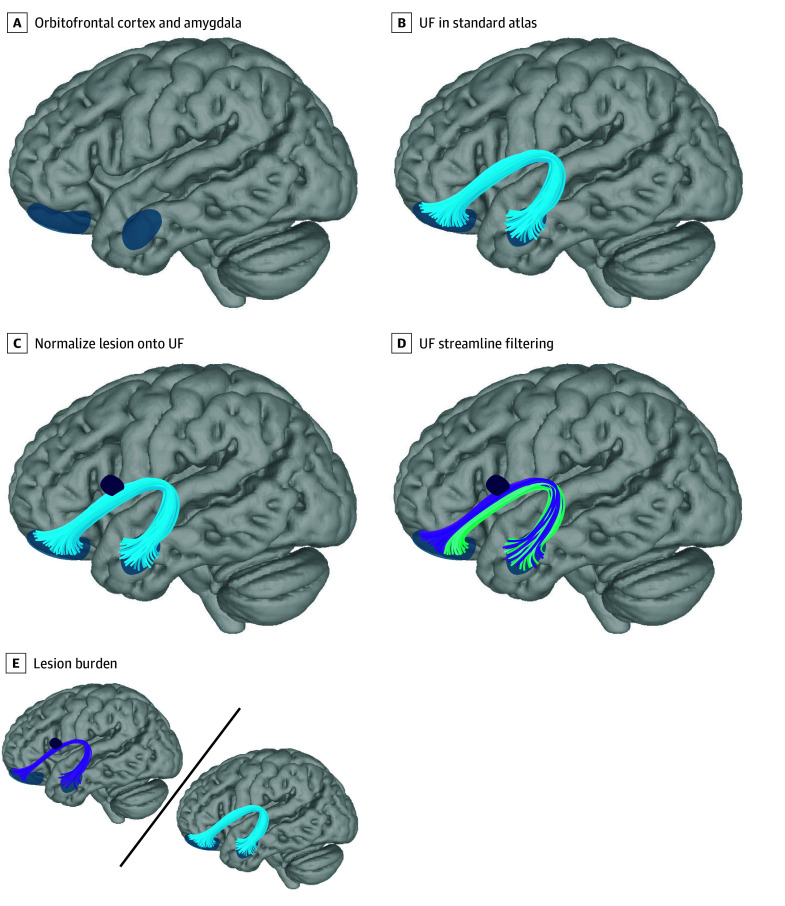
Uncinate Fasciculus (UF) Analysis Pipeline A, The orbitofrontal cortex (OFC) and the amygdala were identified as key brain regions relevant for anxiety and stress circuitry from preclinical and human neuroimaging research.^11,12,13,14,15,16^ B, The UF, which structurally connects the OFC and amygdala, was extracted from a standard atlas.^52^ C, After automated white matter lesion segmentation with the Method for Intermodal Segmentation Analysis, lesions were normalized to the template space of the canonical UF. D, For both right and left UF, streamlines that intersected lesions at any point in their trajectory were considered injured and isolated from the rest of the fascicle. E, Lesion burden was defined as the mean volume occupied by injured streamlines in the UF divided by the mean volume of streamlines in the canonical right and left UF.

To assess the association of white matter lesions with anxiety diagnosis and severity, we performed streamline filtering in DSI Studio.^51,52,53,54^ We first identified the OFC and the amygdala as key brain regions relevant for anxiety and stress circuitry from preclinical and human neuroimaging research (Figure 2A).^16,18,21^ The UF, which structurally connects the OFC and amygdala, was extracted from a standard atlas (Figure 2B).^52^ Next, individual lesion maps were normalized to the template space of the canonical UF (Montreal Neurological Institute 2009b Asymmetric template)^55^ using the T1-weighted–based transform calculated by antsRegistration (Figure 2C).^56,57^ For both the right and left UF, streamlines intersecting lesions at any point in their trajectory were considered injured and isolated from the rest of the fascicle, and the mean volume of streamlines of the left and right side was calculated (Figure 2D). We then created a summary measure of UF disease burden by dividing the mean volume of injured UF streamlines by the mean volume of streamlines in the canonical right and left UF (Figure 2E).

### Statistical Analysis

All analyses were conducted from June 1 to September 30, 2024, with R, version 2024.12.0 (R Project for Statistical Computing). Demographic data, including age, sex, race, PHQ-2 scores, and PROMIS data were compared across anxiety phenotypes with analysis of variance tests for continuous data (age, PHQ-2 score) and χ^2^ tests for categorical variables (sex, race). To test for associations between anxiety subtypes and PROMIS emotional and physical functioning domains, Shapiro-Wilk tests were first used to confirm normality, and then post hoc *t* tests were used. Multiple comparisons were accounted for by controlling the false discovery rate (FDR) (*Q *<* *0.05). We used generalized additive models (GAMs) (mgcv package in R, version 1.8-42)^58^ to test for associations between mean UF burden and anxiety diagnosis (severe anxiety vs without anxiety) as well as associations between UF burden and anxiety severity while controlling for linear and nonlinear age effects. Covariates included sex, total brain volume, and a spline of age. Statistical significance was set at 2-sided *P* < .05 for both hypothesis-driven models.

For all sensitivity analyses, we used GAMs to test for associations between outcome measures and psychopathology, covarying for sex, total brain volume, and nonlinear effects of age and controlling the FDR (*Q *<* *0.05). For our main analyses, we selected the UF as the fascicle of interest given its structural connections between the OFC and amygdala, which are part of a known anxiety circuit. However, there are other structural connections between the prefrontal cortex and the temporal lobe that are functionally connected but subserve different cognitive processes. For example, the fornix connects the prefrontal cortex and hippocampus and is involved in memory.^59^ To test whether our results were specific to anxiety network white matter injury, we evaluated whether anxiety diagnosis and severity were associated with mean fornix burden.

Depression is frequently comorbid with anxiety in healthy populations and is present in up to 50% of patients with MS.^30,60^ To test whether lesion burden in the UF was specific to anxiety severity or associated with general symptoms of both depression and anxiety, we stratified the cohort into those with depression (MS with depression) and those who were psychiatrically asymptomatic (MS without depression) using a previously described phenotyping protocol.^30^ We then tested whether UF burden was associated with a depression diagnosis.

Because it is possible that total lesion volume is associated with anxiety, we tested whether anxiety diagnosis and severity were associated with total lesion volume. To verify if this association was specific to anxiety, we also tested whether total lesion volume was associated with depression. Information about code and instructions for replicating analyses is available in the eMethods in Supplement 1.

## Results

Among the 372 patients included, mean (SD) age was 47.7 (11.4) years; 296 (80%) were female, and 76 (20%) were male. Two patients (1%) were Asian; 76 (20%), Black or African American; 266 (72%), White; 18 (5%), other race; 9 (2%), unknown race; and 1 patient (<1%) declined to answer. Anxiety phenotype stratification produced groups that were balanced for age and sex: MS without anxiety (99 [27%]; mean [SD] age, 49.4 [11.7] years; 74 [75%] female and 25 [25%] male), MS with mild anxiety (249 [67%]; mean [SD] age, 47.1 [11.1] years; 203 (82%) female and 46 [18%] male), and MS with severe anxiety (24 [6%]; mean [SD] age, 47.0 [12.2] years; 19 [79%] female and 5 [21%] male) (Table). Mean (SD) PHQ-2 score was 0.00 (0.00) in the group without anxiety, 0.57 (1.16) in the group with mild anxiety, and 1.16 (1.54) in the group with severe anxiety. Seven of the 372 participants (2%) were prescribed steroids or interferon, which may cause psychiatric adverse effects.

**Table.** Participant Demographics^a^

Characteristic	Participants^b^			*P* value^e^	
	MS without anxiety (n = 99)	MS with mild anxiety (n = 249)^c^	MS with severe anxiety (n = 24)^d^	Anxiety diagnosis	Anxiety severity
Age, mean (SD), y	49.4 (11.7)	47.1 (11.1)	47.0 (12.2)	.36	.22
Sex					
Female	74 (75)	203 (82)	19 (79)	.85	.37
Male	25 (25)	46 (18)	5 (21)		
Race^f^					
Asian	2 (2)	0	0	.07	<.001
Black or African American	19 (19)	54 (22)	3 (13)		
White	68 (69)	180 (72)	18 (75)		
Other	10 (10)	7 (3)	1 (4)		
Declined to answer	0	0	1 (4)		
Unknown	0	8 (3)	1 (4)		
PHQ-2 score, mean (SD)	0.00 (0.00)	0.57 (1.16)	1.16 (1.54)	<.001	<.001

Abbreviations: MS, multiple sclerosis; PHQ-2, 2-question Patient Health Questionnaire.

^a^ All demographic variables were extracted from discrete fields in the electronic medical record.

^b^ Data are presented as number (percentage) of participants unless otherwise indicated.

^c^ Anxiety diagnosis or anxiolytic medication.

^d^ Anxiety diagnosis and anxiolytic medication.

^e^ *P* values reflect analysis of variance tests for continuous data (age, PHQ-2 score) and χ^2^ tests for categorical variables (sex, race). *P* values comparing demographic differences between anxiety diagnosis (MS without anxiety vs MS with severe anxiety) and anxiety severity (MS without anxiety, MS with mild anxiety, and MS with severe anxiety) are displayed.

^f^ Race was patient-reported. “Other” was an option patients could select.

### Association of Anxiety With Physical and Emotional Functioning

**Figure 3.**
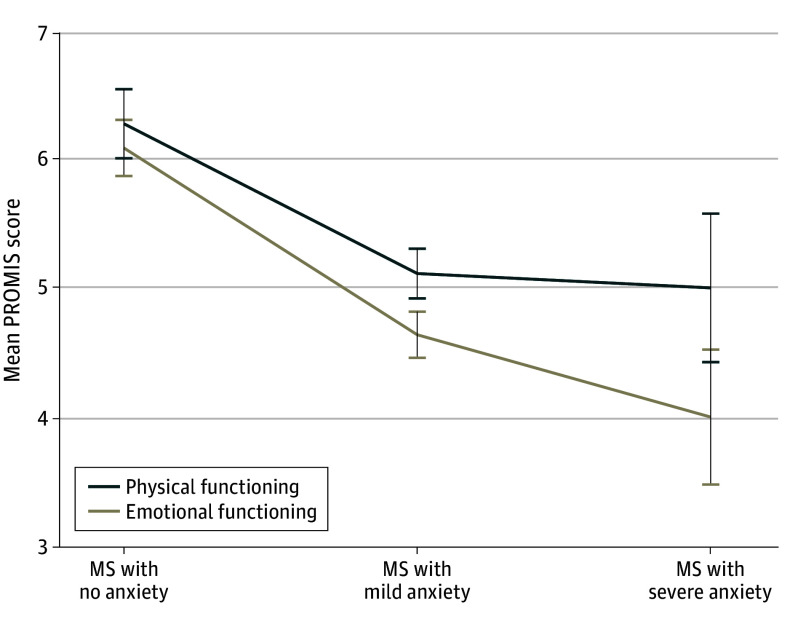
Patient-Reported Outcomes Measurement Information System (PROMIS) Scores and Anxiety Severity The PROMIS assessed current symptom burden in 10 domains, which were stratified into a physical functioning and an emotional functioning summary score. Error bars reflect the SE of the mean.

A subset of participants completed PROMIS scales (eTable 2 in Supplement 1). In 8 of 10 domains, patients who had MS and severe anxiety had worse function than those without anxiety, and worse anxiety severity was parametrically associated with worse functioning. We next compared the association of anxiety diagnosis and anxiety severity with the physical and emotional functioning summary scores (Figure 3). Higher anxiety severity was associated with worse functioning in both the physical and emotional domains, with a larger effect size in the emotional functioning domain (*T* = −4.2 [*P* < .001]; Cohen *f*^2^, 0.28 [95% CI, 0.07-0.67]) than the physical functioning domain (*T* = −2.5 [*P* = .02]; Cohen *f*^2^, 0.10 [95% CI, 0.00-0.33]).

Post hoc *t* tests revealed that compared with MS without anxiety, MS with severe anxiety was associated with worse emotional functioning (*T* = 3.7 [*P* = .02 for FDR]; Cohen *d*, −1.87) (eTable 3 in Supplement 1) but not physical functioning (*T* = 2.0 [*P* = .08 for FDR]). Participants who had MS with mild anxiety and MS with severe anxiety had worse emotional vs physical functioning in paired *t* tests (severe anxiety: *T* = 2.88 [*P* = .047 for FDR]; Cohen *d*, −0.69; mild anxiety: *T* = 2.97 [*P* = .02 for FDR]; Cohen *d*, −0.38), while there were no significant differences in patients with MS without anxiety (*T* = 0.68 [*P* = .51 for FDR]). These findings suggested that severe anxiety symptoms may be dissociable from the psychological impact of physical limitations.

### Association of Anxiety Diagnosis With UF Lesion Burden

**Figure 4.**
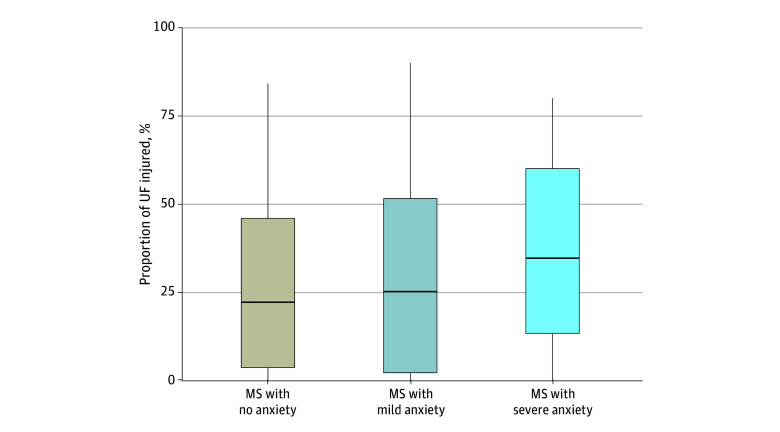
Association of Uncinate Fasciculus (UF) Lesion Burden With Anxiety Severity The horizontal bar inside the boxes indicates the median; lower and upper ends of the boxes, the first and third quartiles; and whiskers, the smallest and largest data points within 1.5 × IQR from the first and third quartiles. MS indicates multiple sclerosis.

We next explored the association between anxiety and UF lesion burden. Anxiety diagnosis (severe vs none) was associated with UF lesion burden (*T* = 2.01 [*P* = .047]; Cohen *f*^2^, 0.19 [95% CI, 0.08-0.52]) (Figure 4). Additionally, we found a parametric association in which greater UF lesion burden was associated with worse anxiety severity (*T* = 2.09 [*P* = .04]; Cohen *f*^2^, 0.10 [95% CI, 0.05-0.21]).

### Specificity of Anxiety-UF Association to Both Fascicle and Psychiatric Phenotype

To confirm that the association between anxiety and UF burden was specific, we first tested whether anxiety diagnosis or severity was associated with fornix injury, and neither of the analyses produced significant results (anxiety diagnosis: *T* = 0.67 [*P* = .50 for FDR]; anxiety severity: *T* = 0.96 [*P* = .41 for FDR]). We next compared UF lesion burden in patients with MS without depression (99 of 306 [32%]) and those with depression (207 of 306 [68%]). Again, no significant association was found (*T* = −1.7 [*P* = .15 for FDR]).

### Association of Total Lesion Volume With General Psychopathology

In addition, we tested for associations of anxiety diagnosis, anxiety severity, and depression diagnosis with total lesion volume. All 3 models showed significant associations (anxiety diagnosis: *T* = 3.07 [*P* = .005 for FDR]; anxiety severity: *T* = 3.22 [*P* = .005 for FDR]; depression diagnosis: *T* = −3.20 [*P* = .005 for FDR]), suggesting that total lesion volume was associated with general psychopathology.

## Discussion

In this study, we developed an EMR phenotyping method to identify anxiety groups among patients with MS and derived a proxy measure of anxiety diagnosis and severity. We found that anxiety in MS was associated with the burden of UF lesions. Specifically, individuals with severe anxiety had a higher UF lesion burden than those without anxiety. We also found a parametric association in which greater anxiety severity was associated with larger UF burden. This association was specific to the UF rather than other prefrontal cortex–temporal white matter tracts and was specific to anxiety and not present in depression.

Previous studies included in a systematic review have sought to disentangle the relationship between anxiety symptoms and MS.^4^ One common hypothesis is that anxiety is a natural psychological consequence of living with a progressive neurological disorder and the associated disability in physical and social functioning.^9^ Others have hypothesized that MS neuropathology alone may underlie anxiety severity.^4^ However, research parsing how lesion location or lesion volume is associated with anxiety has yielded inconsistent results.^7^ In 1 prior study in 48 people with MS, anxiety was associated with white matter lesion load in the fornix.^61^ Another study found no associations.^9^ We attempted to address these gaps by using a large sample of participants with research-quality imaging and performing automated white matter lesion segmentation to decrease cost and time associated with image analysis. By evaluating lesion burden rather than lesion location or volume, we identified a significant association between anxiety and UF lesions. Furthermore, our PROMIS results suggested that physical functioning could be disentangled from emotional functioning in the group with MS and severe anxiety.

Our research adds to the growing body of literature showing that psychopathology in MS is associated with injury in structural pathways that support functional circuits derived from medically healthy populations.^30,62,63^ Our group has previously used white matter lesion network mapping to show that depression in MS is associated with lesions to fascicles that support a functional depression network derived from normative connectome data.^30^ In the current study, we showed that lesions to the UF, the structural connection between brain regions involved in stress circuitry, were associated with anxiety. We also showed that total lesion burden was associated with general psychopathology and was not specific to anxiety, suggesting that both global and circuit-level injury may contribute to anxiety. Prospective studies that evaluate whether UF burden is associated with treatment outcomes are warranted.

### Generalizability and Scalability

To enhance scalability, we introduced an EMR phenotyping framework for assessing anxiety symptom severity that leveraged known physician coding practices and did not require prospective data collection. However, there are limitations to using the EMR for psychiatric phenotyping.^64^ Anxiety is often underdiagnosed in medical populations^34,65^; thus, an *ICD-10* code for an anxiety disorder likely indicates the presence of anxiety symptoms, whereas its absence does not confirm that the person is psychiatrically asymptomatic.^33^ Many medications used for the treatment of anxiety are also approved for the treatment of depression, which presents challenges in defining anxiety phenotypes without prospective assessments.^38,60,66^

We attempted to address these limitations by combining *ICD-10* diagnoses and medications to stratify the anxiety groups and included a mood screen to define psychiatrically healthy comparators. This required making assumptions about how anxiety severity burden manifests in practitioner diagnostic and prescribing behavior, which we could not verify in our study. Though it is possible that the group with severe anxiety represents adequately treated patients with few anxiety symptoms, our PROMIS scores suggested that our phenotyping captured expected patterns described in prospectively acquired samples in which worse anxiety was associated with worse functioning.^67^ We cannot exclude the possibility that comorbid symptoms, including pain or depression, contributed to medication prescriptions in patients with MS who had mild or severe anxiety, though the mean PHQ-2 score in all anxiety groups was less than 3, the positive depression screen threshold.^68^ Psychiatric disease stratification using multilevel EMR phenotyping with external validation may hold promise for EMR research across other psychiatric illnesses, medical populations, and health systems.

### Limitations

Our study has several limitations. Our analysis was based on a lifetime history of anxiety rather than anxiety at the time of the MRI scan. We attempted to mitigate this by validating the anxiety severity groups with PROMIS scores and time-linking them to patients’ most proximal MRI, but they do not reflect psychiatric diagnoses, and the sample was small. Expanded Disability Status Scale scores, a measure of physical disability that is collected in MS clinical trials but is not part of routine care,^69,70^ were not available for participants in our study, though PROMIS scores do quantify physical disability. Prospective studies that combine psychiatric symptom ratings with MS disability assessments and relate them to lesion burden would be informative.

We evaluated the UF in our anxiety analyses based on its anatomic connections with the OFC and amygdala, though UF variability has also been associated with language^71,72^ and memory.^23^ We also found that anxiety was associated with total lesion burden. While our sensitivity analyses suggest that the association between the UF and anxiety may be specific, given the collinearity of UF burden and total lesion burden, we were unable to disambiguate the two. Studies relating lesion burden to prospectively collected neurocognitive and anxiety assessments are required.^73^ Our analyses evaluated structural lesions irrespective of lesion activity. Future studies that compare new lesions and anxiety are warranted.

## Conclusions

In this case-control study, we explored the association between UF lesion burden and anxiety in MS. We developed a multistep EMR phenotyping method to define anxiety groups. We showed that higher UF lesion burden was associated with an anxiety diagnosis and worsening anxiety severity was associated with greater UF burden. This approach holds promise for understanding both anxiety in MS and the role of abnormalities in white matter as a mechanism for anxiety more broadly.

## Notes

Supplement 1. **eTable 1.** Medications Used in the Treatment of Anxiety **eTable 2.** Demographics of Sample With PROMIS Scores **eTable 3.** Post Hoc Analyses of PROMIS Summary Measures
eMethods.

eReferences.

Supplement 2.
Data Sharing Statement
