# Supplementary material for: Uncinate Fasciculus Lesion Burden and Anxiety in Multiple Sclerosis
Source: JAMA Netw Open. 2025 Apr 14;8(4):e254751. doi: 10.1001/jamanetworkopen.2025.4751 (PMC11997724; doi:10.1001/jamanetworkopen.2025.4751)
Supplement: Supplement 1. — eTable 1. Medications Used in the Treatment of Anxiety eTable 2. Demographics of Sample With PROMIS Scores eTable 3. Post Hoc Analyses of PROMIS Summary Measures eMethods. eReferences. [file jamanetwopen-e254751-s001.pdf]

Supplementary Online Content

Baller EB, Luo AC, Schindler MK, et al. Uncinate fasciculus lesion burden and anxiety in multiple sclerosis. *JAMA Netw Open*. 2025;8(4):e254751.
doi:10.1001/jamanetworkopen.2025.4751

eTable 1. Medications Used in the Treatment of Anxiety
eTable 2. Demographics of Sample With PROMIS Scores
eTable 3. Post Hoc Analyses of PROMIS Summary Measures
eMethods.
eReferences.

This supplementary material has been provided by the authors to give readers additional information about their work.

© 2025 Baller EB, et al. *JAMA Network Open*.

eTable 1. Medications used in the treatment of anxiety. Brand and generic medications that are FDA-approved for the treatment of anxiety, as well as medications commonly used off-label for anxiety (e.g. gabapentin, propranolol), are included[1]. Antipsychotic medications were excluded. NaSSA, noradrenergic and specific serotonergic antidepressant; SNRI, serotonin-norepinephrine reuptake inhibitor; SSRI, selective serotonin reuptake inhibitor, TCA, tricylic antidepressant.

	Medication	Class
1	Gabapentin	Anticonvulsant
2	Lyrica	Anticonvulsant
3	Neurontin	Anticonvulsant
4	Pregabalin	Anticonvulsant
5	Atarax	Antihistamine
6	Hydroxyzine	Antihistamine
7	Vistaril	Antihistamine
8	Buspar	Anxiolytic
9	Buspirone	Anxiolytic
10	Alprazolam	Benzodiazepine
11	Ativan	Benzodiazepine
12	Chlordiazepoxide	Benzodiazepine
13	Clonazepam	Benzodiazepine
14	Diazepam	Benzodiazepine
15	Klonopin	Benzodiazepine
16	Librium	Benzodiazepine
17	Lorazepam	Benzodiazepine
18	Oxazepam	Benzodiazepine
19	Serax	Benzodiazepine
20	Valium	Benzodiazepine
21	Xanax	Benzodiazepine
22	Inderal	Beta-blocker
23	Propranolol	Beta-blocker
24	Mirtazapine	NaSSA
25	Remeron	NaSSA
26	Cymbalta	SNRI
27	Duloxetine	SNRI
28	Effexor	SNRI
29	Venlafaxine	SNRI
30	Celexa	SSRI

© 2025 Baller EB, et al. *JAMA Network Open*.

	Medication	Class
31	Citalopram	SSRI
32	Escitalopram	SSRI
33	Fluoxetine	SSRI
34	Lexapro	SSRI
35	Paroxetine	SSRI
36	Paxil	SSRI
37	Prozac	SSRI
38	Sertraline	SSRI
39	Zoloft	SSRI
40	Amitriptyline	TCA
41	Elavil	TCA
42	Nortriptyline	TCA
43	Pamelor	TCA

© 2025 Baller EB, et al. *JAMA Network Open*.

eTable 2. Demographics of sample with Patient-Reported Outcomes Measurement Information System (PROMIS) scores. All demographic variables were extracted from discrete fields in the electronic medical record. Race is patient-reported. Lower PROMIS scores represent more impairment. *P*-values reflect ANOVA tests in continuous data (age, Patient Health Questionairre-2, PROMIS scores) and χ^2 tests for categorical variables (sex, race). *P*-values for demographic differences between anxiety diagnosis (MS+noA vs MS+severeA) as well as P-values for anxiety severity (MS+noA, MS+mildA, MS+severeA) are displayed. MS+noA, MS without anxiety; MS+mildA, MS with mild anxiety (anxiety diagnosis *or* anxiolytic medication); MS+severeA, MS with severe anxiety (anxiety diagnosis *and* anxiolytic medication); SD, standard deviation.

	**MS+noA**	**MS+mildA**	**MS+severeA**	***P*** *Anxiety Diagnosis*	***P*** *Anxiety Severity*
n	12	45	7		
Age (mean [SD])	50.08 [10.30]	49.76 [10.68]	46.29 [8.04]	0.42	0.69
Sex (n [%])				0.43	0.35
Female	7 [75]	38 [84]	7 [100]		
Male	3 [25]	7 [16]	0 [0]		
Race (n [%])				0.98	0.93
Black or African American	2 [17]	11 [24]	2 [29]		
Unknown	0 [0]	1 [2]	0 [0]		
White	10 [83]	33 [73]	5 [71]		
Patient Health Questionnaire-2 (mean [SD])	0.0 [0.0]	1.00 [0.85]	2.00 [2.08]	0.003	0.002
Quality of Life (mean [SD])	4.25 [0.62]	2.93 [1.01]	2.57 [0.98]	<0.001	<0.001
Physical Health (mean [SD])	3.17 [0.58]	2.47 [0.81]	2.29 [0.95]	0.02	0.02
Mental Health and Mood (mean [SD])	3.92 [0.79]	3.40 [0.89]	2.86 [1.21]	0.03	0.05
Social Activities Satisfaction (mean [SD])	4 [0.74]	2.93 [1.27]	2.71 [1.50]	0.02	0.02
Carrying Out Social Activities (mean [SD])	4 [0.85]	3.11 [1.23]	2.29 [1.38]	0.004	0.01

© 2025 Baller EB, et al. *JAMA Network Open*.

Carrying Out Physical Activities (mean [SD])	3.75 [0.87]	3.16 [1.04]	3 [1.00]	0.10	0.16
Emotional Problems (mean [SD])	4.08 [0.51]	3.09 [1.10]	2.71 [1.11]	0.002	0.006
Fatigue Average (mean [SD])	3.75 [0.75]	3.16 [1.19]	3.14 [1.35]	0.22	0.27
Post-Op PROMIS Physical Score (mean [SD])	14.42 [2.15]	11.67 [2.80]	11.57 [3.36]	0.04	0.01
Post-Op PROMIS Mental Score (mean [SD])	16.25 [2.01]	12.36 [3.16]	10.86 [3.58]	0.001	<0.001

© 2025 Baller EB, et al. *JAMA Network Open*.

eTable 3. Post hoc analyses of Patient-Reported Outcomes Measurement Information System (PROMIS) summary measures. Pairwise contrasts are reported as *P*-values with corresponding Cohen's *d*. Multiple comparisons were accounted for by controlling the False Discovery Rate (*Q*<0.05). MS+noA, MS without anxiety; MS+mildA, MS with mild anxiety (anxiety diagnosis *or* anxiolytic medication); MS+severeA, MS with severe anxiety (anxiety diagnosis *and* anxiolytic medication).

	T	P_{fdr}	Cohen's *d*
Physical vs Emotional Functioning			
MS+noA	0.68	0.51	-
MS+mildA	2.97	0.02	-0.38
MS+severeA	2.88	0.047	-0.69
Emotional Functioning<br>MS+severeA vs MS+noA	3.68	0.02	-1.87
Physical Functioning<br>MS+severeA vs MS+noA	2.00	0.96	-

© 2025 Baller EB, et al. *JAMA Network Open*.

eMethods

Code and instructions for replicating all analyses can be found at: https://baller-lab.github.io/msanxiety/.

© 2025 Baller EB, et al. *JAMA Network Open*.

eReferences

1. Garakani A, Murrough JW, Freire RC, et al. Pharmacotherapy of Anxiety Disorders: Current and Emerging Treatment Options. *Front Psychiatry*. 2020;11:595584. doi:10.3389/fpsyt.2020.595584

2. List of 70 Multiple Sclerosis Medications Compared. Drugs.com. Accessed September 12, 2024. https://www.drugs.com/condition/multiple-sclerosis.html

© 2025 Baller EB, et al. *JAMA Network Open*.
