# Supplementary material for: Uncinate Fasciculus Lesion Burden and Anxiety in Multiple Sclerosis
Source: JAMA Netw Open. 2025 Apr 14;8(4):e254751. doi: 10.1001/jamanetworkopen.2025.4751 (PMC11997724; doi:10.1001/jamanetworkopen.2025.4751)
Supplement: Supplement 2. — Data Sharing Statement [file jamanetwopen-e254751-s002.pdf]

Data Sharing Statement

Baller. Uncinate Fasciculus Lesion Burden and Anxiety in Multiple Sclerosis. *JAMA Netw Open*. Published April 14, 2025. doi:10.1001/jamanetworkopen.2025.4751

Data
Data available: No

Additional Information
Explanation for why data not available: All analytic code will be shared via github. Unfortunately, given that this study used data obtained via the electronic medical record and is HIPAA protected, we are not able to share the raw data.
